# Associations Between Sleep Satisfaction and Physical Activity Rates Among Secondary Medical Areas in Japan: An Ecological Study

**DOI:** 10.7759/cureus.72814

**Published:** 2024-11-01

**Authors:** Junko Nishimura, Mayumi Mizutani

**Affiliations:** 1 Department of Integrated Health Sciences, Graduate School of Medicine, Nagoya University, Nagoya, JPN

**Keywords:** daily living activities, ecological study, fast walking speed, japan, regular exercise, sleep satisfaction

## Abstract

Objective

Insomnia is a common problem in Japan and other countries, indicating the need for effective strategies to improve sleep quality. Although the associations between specific exercises, physical activity, and sleep satisfaction have been clarified, few reports have examined the associations with normal lifestyle habits. This study aims to clarify the associations between sleep satisfaction and physical activity (i.e., regular exercise, daily living activities, and fast walking speed) at secondary medical area levels in Japan.

Methodology

This ecological study utilizes the 2021 National Database of Health Insurance Claims and Specific Health Checkups of Japan (NDB) open data managed by the Japanese Ministry of Health, Labour, and Welfare across 335 Japanese secondary medical areas. The data were analyzed using the multiple regression analysis forced entry method, in which the percentages of sleep satisfaction and physical activity were the objective and explanatory variables, respectively.

Results

Data from 335 medical areas and a total of 30.39 million individuals, comprising 16.49 million males and 13.90 million females, were analyzed. Among the 335 medical areas, the mean sleep satisfaction percentages were 70.8% for males and 67.0% for females. The mean percentages for three physical activities were (1) regular exercise for 31.8% of males and 25.3% of females, (2) daily living activities for 43.2% of males and 43.7% of females, and (3) fast walking speed for 46.8% of males and 43.4% for females. The sleep satisfaction percentage was negatively associated with regular exercise in males aged 70-74 (B = −0.10, p = 0.029). Significant positive associations were found for daily living activities in males aged 40-49 (B = 0.24, p < 0.001), 50-59 (B = 0.14, p = 0.023), and 60-69 (B = 0.09, p = 0.024) and in females aged 40-49 (B = 0.12, p = 0.001) and 50-59 (B = 0.08, p = 0.036). Positive associations were also found for fast waking speed in males aged 60-69 years (B = 0.12, p = 0.029) and 70-74 (B = 0.24, p < 0.001) and in females aged 70-74 (B = 0.16, p = 0.001).

Conclusions

This study revealed that the factors positively associated with sleep satisfaction include daily living activities and walking speed, which vary by gender and age group. Tailored daily lifestyle health guidance is needed to improve sleep quality across various demographics.

## Introduction

Quality sleep, characterized by a sufficient duration and genuine sense of restfulness, is critical to maintaining and improving mental and physical health. However, previous studies have found that sleep disorders, such as insomnia, are widespread globally, including in Japan. For example, in the United States, 50-70 million adults suffer from sleep disorders [1], and in South Korea, the sleep disorder prevalence doubled from 7.6% to 14.4% over a 10-year period from 2011 to 2020 [2]. In a 2016 nationwide epidemiological survey of Japan, the prevalence of insomnia was recorded at 12.2% for males and 14.6% for females; this prevalence was found to increase with age and was higher in females [3]. Furthermore, sleep disorders are closely related to lifestyle-related diseases, such as depression and metabolic syndrome [4,5]. Therefore, implementing measures to improve sleep quality is imperative to maintain civilian health and prevent deterioration in overall quality of life.

Since sleep satisfaction, which is a subjective measure independent of sleep duration, is important for good health, research focused on improving sleep quality is receiving increased attention. Previous sleep quality studies have concluded that healthy lifestyles, including nutritionally balanced diets and increased physical activity, can promote good sleep [6-8]. In particular, as physical activity can increase fatigue, adequate and quality sleep is extremely crucial. Physical activities have been broadly classified into two, namely, exercises, which are needed to maintain and improve physical fitness, and daily living activities, including daily work, household chores, and hobbies. Furthermore, aerobic exercise, such as walking and jogging, has been reported to increase sleep duration and the sense of sleep rest [9], and strength training using dumbbells has been shown to improve sleep [10]. Meanwhile, sedentary behavior has been associated with an increased risk of insomnia and sleep disturbances [11].

As most previous studies have focused on planned exercises or specific lifestyle activities, few have examined the usefulness of casual, everyday lifestyle habits. Further, as many studies tend to examine study populations comprised of people with insomnia and other clinical sleep problems, it has been difficult to identify the trends in people who get satisfactory sleep. Insomnia symptoms increase with age, with more than half the people aged 60 or older experiencing them; therefore, to develop preventive support, research focusing on the general population aged 40 and older is needed [12].

Therefore, this study aimed to determine the associations between sleep satisfaction and physical activities in the general population aged 40 and older by gender and age group.

## Materials and methods

Study design and data sources

This was an ecological study based on secondary data obtained from 335 Japanese secondary medical areas of the National Database of Health Insurance Claims and Specific Health Checkups of Japan (NDB) FY2021 open data maintained by the Ministry of Health, Labour, and Welfare (hereafter, NDB data). The term "medical areas" refers to a geographic region designated for healthcare services; these areas are categorized as primary, secondary, or tertiary based on the type of healthcare services provided. Compared with primary medical areas, secondary ones encompass regional hospitals that offer services such as hospitalization [13]. The Ministry of Health, Labour, and Welfare collects data via surveys completed by individuals who undergo specific health checkups in Japan. The specific health checkup is an annual nationwide initiative in the Japanese Health Insurance System for the prevention, detection, and intervention of lifestyle-related diseases in enrollees aged 40-74 years. For the FY2021 survey, 30.39 million individuals participated in specific health checkups, representing 56.5% of the population aged 40-74 years [14]. Of the participants, 16.49 million were males, and 13.90 million were females. Moreover, 10.10, 9.78, 6.78, and 3.73 million participants were aged 40-49, 50-59, 60-69, and 70-74 years, respectively. Therefore, the sample size was sufficiently large to obtain a fairly accurate representation of the health trends of Japanese individuals who undergo these specific health checkups. In these checkups, people between 40 and 74 are required to complete a standard questionnaire, which includes lifestyle questions, such as sleep, regular exercise, daily activities, and walking speeds, the questions for which are answered with a yes or a no. This standard questionnaire has been developed by the Ministry of Health, Labour, and Welfare and is available on their website [15].

In this study, the objective variable was the percentage of respondents who answered yes to the question "I am well-rested from sleep" (hereinafter referred to as sleep satisfaction). The explanatory variables included (1) the percentage of respondents who answered yes to the question "I engage in light sweaty exercise for 30 minutes or more per session, at least 2 days per week, for at least one year" (hereinafter referred to as regular exercise); (2) the percentage of respondents who answered yes to the question "I walk or engage in similar physical activity in my daily life for at least one hour per day" (hereinafter referred to as daily living activities); and (3) the percentage of respondents who answered yes to the question "I walk faster than others of the same sex at about the same age" (hereinafter referred to as fast walking speed). All data were organized by gender (i.e., male and female) and age group (40-49, 50-59, 60-69, and 70-74).

Statistical analysis

First, the means, standard deviations, and maximum and minimum values for the responses from 335 secondary medical areas were calculated for each assessment item related to sleep and physical activity by gender and age group. Subsequently, to explore the factors associated with sleep and physical activity, a multiple regression analysis using the forced entry method was employed for which sleep satisfaction and all the types of physical activity (i.e., regular exercise, daily living activities, and fast walking speed) were the objective and explanatory variables, respectively. Moreover, the variance inflation factor was calculated to detect problems of multicollinearity between the explanatory variables. The cutoff value of the variance inflation factor was <5. IBM SPSS Statistics for Windows, Version 28 (Released 2021; IBM Corp., Armonk, New York) was employed for all statistical analyses, with the statistical significance set at 5%.

Ethical considerations

The data used were publicly available and did not include any personally identifiable information. Therefore, ethical permission was not required for this study.

## Results

Sleep satisfaction and physical activity characteristics

The mean sleep satisfaction percentage was 70.8% for males and 67.0% for females. Males aged 70-74 reported the highest sleep satisfaction, and females aged 50-59 reported the lowest. The average percentage of regular exercise was 20%-30%. The highest regular exercise percentage was reported by males aged 70-74 (44.7%) and the lowest by females aged 40-49 (13.9%). The highest percentage of engagement in daily living activities was reported by females aged 70-74 (52.9%). Moreover, the fastest walking speed was reported by males aged 70-74 (50.6%) (Table 1).

**Table 1:** Sleep satisfaction and physical activity characteristics Sleep satisfaction: the percentage of respondents from 335 secondary medical areas who answered yes to the question "I am well-rested from sleep." Regular exercise: the percentage of respondents who answered yes to the question "I engage in light sweaty exercise for 30 minutes or more per session, at least 2 days per week, for at least one year." Daily living activities: the percentage of respondents who answered yes to the question "I walk or engage in similar physical activity in my daily life for at least one hour per day." Fast walking speed: the percentage of respondents who answered yes to the question "I walk faster than others of the same sex at about the same age."

Characteristics	Male				Female			
	Mean	SD	Max	Min	Mean	SD	Max	Min
Sleep satisfaction (%)								
40–49 years	66.3	3.7	78.4	50	63.3	3.1	71.9	48
50–59 years	65.3	3.8	77.3	49	59.9	3.2	71.6	47
60–69 years	72.5	3.4	84	59	69.3	3.2	85.3	52
70–74 years	79.2	3.6	89.1	58	75.4	3.8	91.4	52
Total	70.8	6.6	89.1	49	67	6.8	91.4	47
Regular exercise (%)								
40–49 years	25.7	2.6	38.1	18	13.9	2.3	22.9	7.2
50–59 years	25.3	3.3	39.2	16	17.7	3.1	27	9.5
60–69 years	31.5	4.1	44.4	17	28.3	4.2	36.3	13
70–74 years	44.7	5.8	55.7	17	41.4	5.4	51.6	15
Total	31.8	8.9	55.7	16	25.3	11	51.6	7.2
Daily living activities (%)								
40–49 years	42.5	3.9	63.4	29	37.9	5.2	62.9	18
50–59 years	36.7	3.9	57.4	25	38.7	5.7	63.5	17
60–69 years	41.6	4.9	60	26	45.2	6.9	73.2	24
70–74 years	52.2	6.1	73.1	28	52.9	7.9	83.6	24
Total	43.2	7.4	73.1	25	43.7	8.9	83.6	17
Fast walking speed (%)								
40–49 years	43.9	4.1	56.6	30	35.7	4.3	50.3	22
50–59 years	45.5	3.9	57.2	31	41.2	4.6	57.1	25
60–69 years	47.2	4.4	59.6	26	47.2	5.3	64.1	24
70–74 years	50.6	5.2	68.6	23	49.4	5.5	72.7	22
Total	46.8	5.1	68.6	23	43.4	7.3	72.7	22

Relationship between sleep satisfaction and physical activity

Table 2 shows the multiple regression analysis results in which the sleep satisfaction percentage was the objective variable and regular exercise, daily living activities, and fast walking speed were the explanatory variables. A negative significant relationship for regular exercise was found in males aged 70-74 years old; that is, a 1% higher percentage for regular exercise was associated with a 0.10% lower percentage in males aged 70-74 who perceived their sleep rest to be adequate. The association was significant for daily living activities in males aged 40-49, 50-59, and 60-69 and for females aged 40-49 and 50-59. The strongest association was found in males aged 40-49 years, where a 1% higher percentage of daily living activities was associated with a 0.24% increase in sleep satisfaction. There was a significant association between fast walking speed and sleep satisfaction for males aged 60-69 and 70-74, as well as females aged 70-74. The strongest association was found in males aged 70-74, where a 1% higher percentage of fast walking speed was associated with a 0.24% increase in sleep satisfaction.

**Table 2:** Relationship between sleep satisfaction and physical activity Multivariate regression model Objective variable: sleep satisfaction (the percentage of respondents from 335 secondary medical areas who answered "yes" to the statement "I feel well-rested after sleeping.") Explanatory variables: regular exercise (the percentage of respondents who answered "yes" to the statement "I have engaged in light, sweat-inducing exercise for 30 minutes or more at a time at least 2 days per week for at least 1 year."); daily living activities (the percentage of respondents who answered "yes" to the statement "I walk or engage in similar physical activity in my daily life for at least 1 hour per day.”); and fast walking speed (the percentage of respondents who answered "yes" to the statement "I walk faster than others who are of the same sex and approximately the same age as me.") B: unstandardized regression coefficient; p: significance; R^2^: coefficient of determination; VIF: variance inflation factor

Age Group	Physical Activity	Male				Female			
		B	p	R^2^	VIF	B	p	R^2^	VIF
Age 40–49	Regular exercise	0.16	0.065	0.08	1.3	0.05	0.610	0.07	1.6
	Daily living activities	0.24	< 0.001		1.2	0.12	0		1.3
	Fast walking speed	–0.01	0.842		1.3	0.07	0.15		1.5
Age 50–59	Regular exercise	−0.09	0.228	0.02	1.5	−0.10	0.18	0.02	1.8
	Daily living activities	0.14	0.023		1.3	0.08	0.04		1.4
	Fast walking speed	0.08	0.19		1.5	0.08	0.15		1.8
Age 60–69	Regular exercise	−0.11	0.052	0.03	1.7	−0.06	0.33	0.00	1.9
	Daily living activities	0.09	0.024		1.2	0.04	0.12		1.2
	Fast walking speed	0.12	0.029		1.7	0.04	0.43		2.0
Age 70–74	Regular exercise	−0.10	0.029	0.08	2.0	−0.07	0.17	0.03	1.8
	Daily living activities	0.05	0.098		1.1	0.04	0.2		1.1
	Fast walking speed	0.24	<0.001		2.0	0.16	0		1.8

## Discussion

This study's analysis of 335 medical areas revealed that physical activity (regular exercise, daily living activities, and fast walking speed) was related to sleep satisfaction.

First, the analysis revealed that 70.8% of males and 67.0% of females were feeling well-rested after sleep. Another national survey conducted in 2018 by the Ministry of Health, Labour, and Welfare revealed that 80.6% of males and 80.1% of females aged 40 and over were well-rested and fairly well-rested after sleep [16]. Therefore, the data used in this study showed a slightly higher percentage of respondents who were dissatisfied with their sleep. However, the finding that the percentage of respondents who were getting good sleep increased with age was similar to the results from another survey of 3,000 people conducted in 2022 [17]; therefore, the NDB data used in this study was considered representative of the current sleep characteristics in Japan.

The relationship between percentages of sleep satisfaction and fast walking speed was significant in males aged 60-69 and 70-74 and for females aged 70-74. Therefore, we inferred from these results that those who maintained the muscle mass to walk fast, even at an older age, had better sleep quality. Research on walking speed and sleep quality has been somewhat limited. However, a cross-sectional study of community-dwelling older adults aged 80 years or older reported that poor sleep quality was associated with decreased muscle strength and slower walking speed [18]. Therefore, further evidence is needed in more focused studies that examine whether specific walking speeds can improve sleep quality and assess whether there is a relationship between sleep quality and muscle mass.

Significant associations were found between daily living activities and sleep satisfaction for both males and females aged 40-49 and 50-59, which suggests that people who are more active because of activities such as work and child-rearing are more satisfied with their sleep. The results in Table 1 show that the daily living activity percentage for people aged over 60 is approximately 40%-50%, which indicates that health guidance is necessary to increase daily physical activity, especially for people over 60.

A significant negative relationship was found between sleep satisfaction and regular exercise in males aged 70-74 years old, which suggests that regular exercise had an adverse effect on sleep satisfaction in this age group. A national internet survey of 2,000 people conducted in 2023 found that the higher the age, the longer the daily exercise time, with 29.5% of males in their 70s exercising for one hour and 12.5% for two to three hours [19]. Therefore, males over 60 may experience poor sleep because they overexercise, and exercise time health guidance should be given to ensure that older people do not become overly fatigued.

This study has four limitations. First, the NDB open data used in this study included only people who had received specified health checkups; therefore, no data were included for people who did not receive these checkups. Although 53.8 million people were eligible for the specified health checkups in FY2021, only 30.39 million, or 56.5%, attended these checkups [14]. According to a questionnaire survey of those who had not taken the specified health checkup, the most common reasons for not taking the checkup were "busy with work and household chores" for people aged 49 or younger and "attending a hospital" for those aged 50 or older [20], suggesting that the analyzed data have little influence on social roles and health issues. Second, the questionnaire responses were only cross-sectional; therefore, causal relationships could not be confirmed. Third, as the objective variable was binary (indicating sleep satisfaction as either yes or no), the exact factors contributing to sleep issues may not be fully captured. In addition, sleep dissatisfaction in the general population constitutes multiple subfactors, including short sleep duration, difficulty falling asleep, and frequent nocturnal awakening [21]. Future studies should investigate the potential relationships between these subfactors and physical activity. Fourth, as this study was an ecological study, an ecological fallacy is possible. In the future, additional individual-level studies are needed to better understand the relationships between sleep quality and physical activity.

## Conclusions

This ecological study revealed the associations between sleep satisfaction and physical activity in secondary medical areas in Japan by gender and age group. The factors associated with being well-rested from sleep were fast walking speed for males in their 60s and males and females in their early 70s. A higher percentage of males and females in their 40s and 50s who had regular daily living activities also had higher sleep satisfaction. However, for males in their early 70s, higher than normal regular exercise was associated with lower sleep satisfaction. Overall, to improve sleep quality, health guidance focused on gender and age-group-specific characteristics is required.
